# Oldest preserved umbilical scar reveals dinosaurs had ‘belly buttons’

**DOI:** 10.1186/s12915-022-01329-9

**Published:** 2022-06-07

**Authors:** Phil R. Bell, Christophe Hendrickx, Michael Pittman, Thomas G. Kaye

**Affiliations:** 1 School of Environmental and Rural Science, University of New England, Armidale, NSW Australia; 2 Unidad Ejecutora Lillo, CONICET-Fundación Miguel Lillo, Miguel Lillo, San Miguel de Tucumán, Tucumán, Argentina; 3 School of Life Sciences, The Chinese University of Hong Kong, Shatin, Hong Kong SAR China; 4 Department of Earth Sciences, University College London, London, UK; 5 Foundation for Scientific Advancement, Sierra Vista, AZ USA

**Keywords:** Ceratopsia, *Psittacosaurus*, Umbilicus, Cretaceous, Mesozoic, Development

## Abstract

**Background:**

In egg-laying amniotes, the developing embryo is tethered to a number of the extraembryonic membranes including the yolk sac and allantois that deliver oxygen and nutrients and remove metabolic waste products throughout embryonic development. Prior to, or soon after hatching, these membranes detach from the animal leaving a temporary or permanent umbilical scar (umbilicus) equivalent to the navel or ‘belly button’ in some placental mammals, including humans. Although ubiquitous in modern mammals and reptiles (including birds), at least early in their ontogeny, the umbilicus has not been identified in any pre-Cenozoic amniote.

**Results:**

We report the oldest preserved umbilicus in a fossil amniote from a ~130-million-year-old early-branching ceratopsian dinosaur, *Psittacosaurus*. Under laser-stimulated fluorescence (LSF), the umbilicus is revealed as an elongate midline structure delimited by a row of paired scales on the abdomen. The relatively late ontogenetic stage (close to sexual maturity) estimated for the individual indicates that the umbilicus was probably retained throughout life.

**Conclusions:**

Unlike most extant reptiles and birds that lose this scar within days to weeks after hatching, the umbilicus of *Psittacosaurus* persisted at least until sexual maturity, similar to some lizards and crocodylians with which it shares the closest morphological resemblance. This discovery is the oldest record of an amniote umbilicus and the first in a non-avian dinosaur. However, given the variability of this structure in extant reptilian analogues, a persistent umbilical scar may not have been present in all non-avian dinosaurs.

**Supplementary Information:**

The online version contains supplementary material available at 10.1186/s12915-022-01329-9.

## Background

Amniotes are characterised by the presence of a number of extraembryonic membranes (allantois, amnion, chorion and yolk sac) and a semipermeable eggshell that provide a stable environment and nourishment for the developing embryo [1–3]. Two of these membranes in particular—the allantois and yolk sac—are intimately connected to the embryo via vitelline and allantoic blood vessels that penetrate the abdominal wall and, in placental mammals, are enclosed within a long umbilical cord [1, 2]. Immediately prior to or soon after hatching, this communication is severed and the yolk sac is internalised [1, 2], although the opening in the abdominal wall may take several days to weeks to fully close, leaving an umbilical scar or umbilicus [3]. Detachment of the placental mammal umbilical cord after birth results in the characteristic navel or ‘belly button’, which is the topographic and developmental equivalent of the umbilicus in reptiles and birds.

In the fossil record, exceptionally preserved soft tissues provide an invaluable lens through which to study the appearance, biology and ecology of extinct organisms, augmenting data from more commonly preserved hard parts, including the bones and exoskeletons [4, 5]. A nearly complete individual of the early-branching ceratopsian *Psittacosaurus* (SMF R 4970, housed at the Senckenberg Research Institute and Natural History Museum Frankfurt, Frankfurt, Germany) from the Early Cretaceous Jehol Group of Liaoning Province, China [6] is an exemplar of the remarkable insights that exceptionally-preserved dinosaur soft tissues have revealed. The individual in SMF R 4970 is preserved lying on its back and is almost entirely shrouded by the preserved integument, including epidermal scales, the keratinous jugal ‘horn’ and a long plume of tail bristles [6, 7]. Exceptional preservation has also permitted prior identification of its cloaca—the first occurrence in a non-avian dinosaur [8]—as well as skin pigmentation patterns and evidence of whole-body countershading [9]. Here, we document the anatomy of the umbilical scar of specimen SMF R 4970, which is newly identified here based on the first-hand study of the specimen under laser-stimulated fluorescence (LSF) imaging and a survey of umbilical scarring in extant lepidosaurs and archosaurs.

## Results

Scales in *Psittacosaurus* are strongly regionalised across the body. Aspects of this have been described in SMF R 4970 previously [6, 8–10], and a full description of the integument of SMF R 4970 will be given by the authors elsewhere. Here, we summarise these studies and our own observations. The pectoral girdle and limbs are covered in typically small ($\overline{x}$ diameter = 1.9 mm) polygonal (3–6-sided) basement scales with larger (diameter = 3.1 mm) diamond-shaped scales surrounding the ankle. Large, truncated-cone-shaped feature scales ($\overline{x}$ diameter = 8.8 mm; up to 6.8 mm high) are irregularly distributed across the girdle region and upper (proximal) half of the brachium. On the flanks, basement scales are anteroposteriorly elongated and roughly diamond-shaped (~ 2.3 × 1.7 mm) with a small number of larger circular-to-irregular feature scales (~ 3–4 mm diameter). Minute reticulate scales ($\overline{x}$ diameter = 0.5 mm) cover the palmar surface of the manus whereas they are larger ($\overline{x}$ diameter = 1.2 mm) and ovoid on the plantar surface of the pedes. The tail (excluding the cloaca and ischial callosity) consists of vertical bands (mediolaterally oriented in life) of typically rounded-quadrangular scales that range from 1.7 to 3.3 mm in height and generally increase in size posteriorly.

**Fig. 1:**
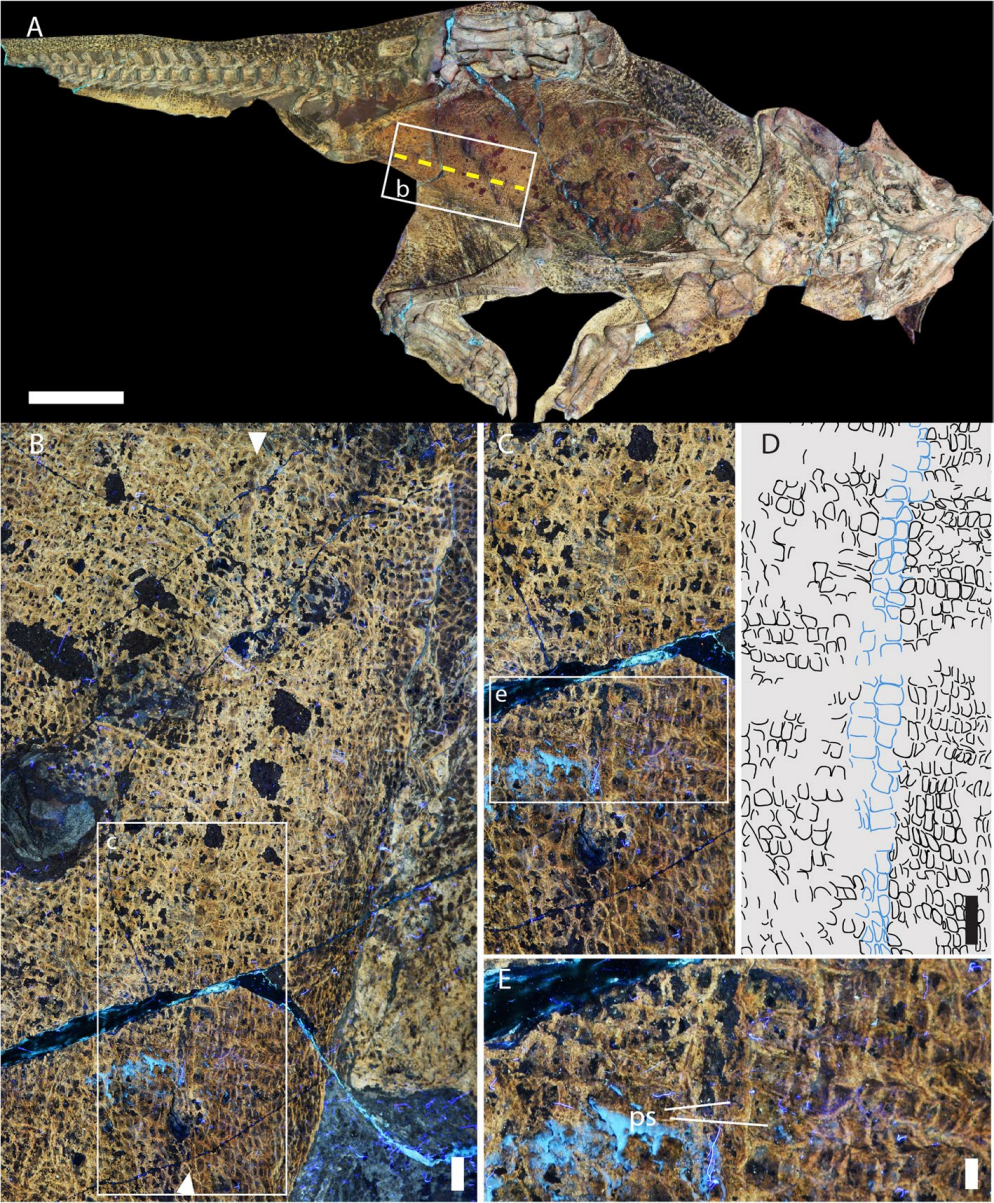
Umbilical scar in *Psittacosaurus* SMF R 4970 under LSF*.* **A** Cropped image of *Psittacosaurus* sp. (SMF R 4970) showing just the skeleton and soft tissue outlines, with the umbilical scar highlighted by the dashed yellow line. **B** Close up of boxed region in (**A**) with the maximal anteroposterior extent of the umbilical scar indicated by arrowheads. Wrinkling forming irregular wavy creases in the integument can be seen on the far right on this image where the abdomen meets the inner thigh; **C**, **D** Close up of boxed region in (**B**) showing paired quadrangular scales (blue outline in **D**) delimiting the umbilicus. Transverse banding is visible in the remaining abdominal scales (black outlines in **D**). **E** Close up of paired quadrangular scales (ps). A clear line of interstitial tissue, delimiting the former scar, can be seen between the paired scales. Anterior is towards the top in (**B**–**E**). Scale bars equal 5 mm (**B**–**D**) and 2 mm (**E**)

**Fig. 2:**
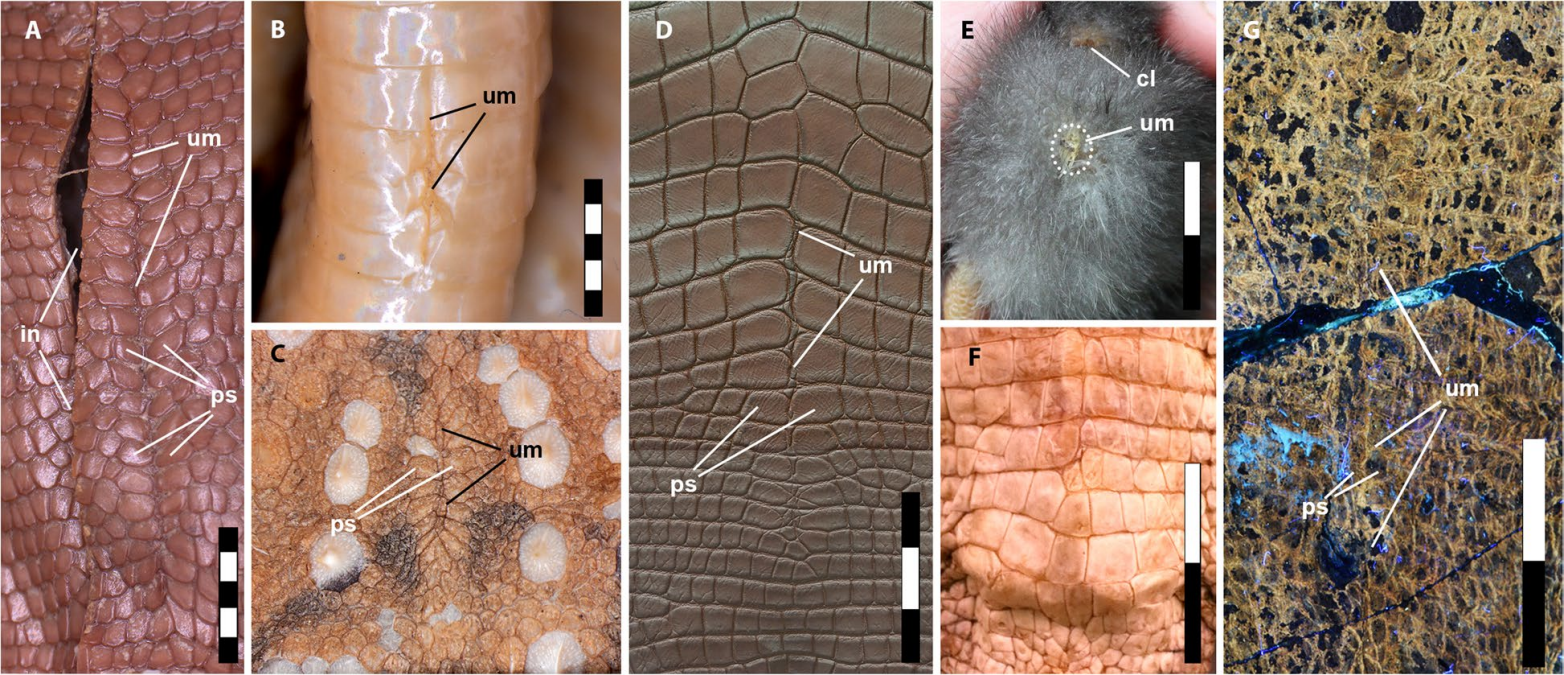
Umbilical scars in modern reptiles, birds and *Psittacosaurus.* **A** Spiny-tailed lizard (*Uromastix* sp.), adult, snout-vent length (SNV) = 168 mm; **B** eastern brown snake (*Pseudonaja textilis*), hatchling, SNV = 239 mm; **C** thorny devil (*Moloch horridus*), adult, SNV = 95 mm; **D** freshwater crocodile (*Crocodylus johnsoni*), age unknown (photo: Ohmega1982/Shutterstock.com); **E** domestic chicken (*Gallus gallus domesticus*), 3-day-old chick (photo: A. Moss, used with permission); **F** freshwater crocodile (*Crocodylus johnsoni*) lacking umbilical scar, hatchling, SNV = 288 mm; **G** *Psittacosaurus* SMF R 4970. **A**–**F** Under white light and **G** under LSF. Abbreviations: cl, cloaca; in, incision made during dissection; ps, paired umbilical scales; um, umbilicus. Scale bars equal 5 mm (**A**–**C**) and 3 cm (**D**–**E**)

Illumination of the specimen under LSF reveals the squamation patterns in extraordinary detail and permits the identification of individual scales [7, 9]. In particular, the soft underparts of the animal, between the rib cage and extending posteriorly between the ischia, are covered by small ($\overline{x}$ length = 1.4 mm) quadrangular scales arranged into distinct transverse ‘bands’ as is typical of modern crocodylians and some squamates (e.g., spiny-tailed lizards *Uromastix* spp.; Figs. 1 and 2). In SMF R 4970, this transverse banding is restricted to the soft abdomen, as well as the ventral surface of the tail, although the quadrangular scales there are much larger (up to 3.3 mm in dorsoventral height). Extending anteriorly from the ischial callosity [7, 9], the transverse bands are broken along the ventral midline of the animal by a distinct longitudinal row of paired quadrangular scales (Fig. 1). These paired scales are often larger ($\overline{x}$ length = 2.5 mm; Additional file 1: Table S1) or of similar size to the surrounding abdominal scales, uniformly distributed with regular margins and upper surfaces, and extend in a line from just in front of the ischiadic symphysis anteriorly for ~13 cm, equivalent to ~14% of the snout-vent length (SVL = 74 cm). The interstitial tissue between the paired scales forms an obvious linear feature even where the paired scales more closely match the size and shape of the remaining abdominal scales (e.g., in the anterior part of the midline structure; see the upper half of the image in Fig. 1B, C). Such a clear linear (anteroposterior) demarcation between scales is not present anywhere else on the specimen. There is no indication of wrinkling or overlapping of scales in this part of the abdomen that would suggest taphonomic or other distortion of the skin. Wrinkling of the integument is present in other parts of the body such as the cloaca and limbs. These invariably show scales that are truncated or distorted (as a result of the two-dimensional projection of the originally curved, folded surface) and have sharp, conspicuous boundaries where the integument is overlapping. In contrast, the posterior part of the abdominal skin appears taut with no evidence of wrinkling except where it meets the hindlimbs, which are far from the midline (Fig. 2A). The interstitial tissue between the paired quadrangular scales and between the surrounding abdominal scales is uniformly narrow with no evidence of stretching, nor is there evidence of distortion, truncation, or conspicuous boundaries indicative of folding/wrinkling of the skin.

Histological analysis will not be allowed by the museum due to the rarity and historical nature of the specimen. Thus, we used femoral length to estimate the age of the individual. The right femur of SMF R 4970 is ~140mm long, which is similar to the femoral lengths of *P. lujiatunensis* IVPP V12617 (138mm) and IVPP V18344 (145mm), LPM R00128 (135mm) and R00138 (144mm), PKUVP V1053 (149mm), and IVPP V1056 (135mm), which all belong to ~6–7-year-old subadults (see Table 1 and Fig. 5 of Erickson et al. [11] and Supplementary table S2 of Zhao et al. [12]). This age is just shy of sexual maturity and at the beginning of the exponential growth phase (see table 1 and fig. 5 of Erickson et al*.* [11] and Supplementary table S2 of Zhao et al*.* [12]). The femoral length of SMF R 4970 is therefore the closest match to a nearly sexually mature subadult (see table 1 and fig. 5 of Erickson et al. [11] and Supplementary table S2 of Zhao et al. [12]).

## Discussion

**Fig. 3:**
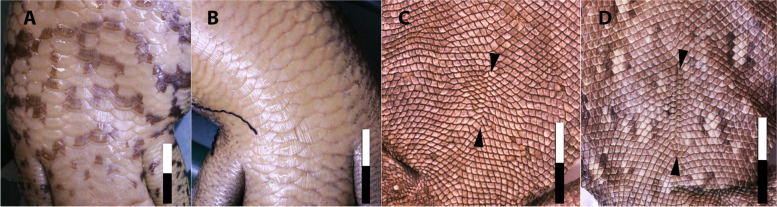
Variation in umbilical scarring in extant lizards. Uninterrupted abdominal scales with no umbilical scarring in (**A**). The blue-tongued lizard (*Tiliqua scincoides*) and **B** the land mullet (*Bellatorias major*). Poorly demarcated umbilicus (endpoints indicated by arrowheads) without distinctive paired scales in (**C**). The bearded dragon (*Pogona barbata*) and **D** the eastern water dragon (*Amphibolurus barbartus*). Anterior is up in all images. Scale bar increments in centimetres

The midline structure in SMF R 4970 consisting of paired scales has the shape and restricted position to the posterior part of the abdomen commensurate with the morphology of the umbilicus (umbilical scar) in extant amniotes. Unlike most mammals, reptiles (including birds) do not have a true umbilical cord—which is an elongate allantois-derived structure linking the placenta and the embryo [13]. Instead, the reptilian embryo is in direct communication with the yolk sac and allantois (or chorioallantoic membrane [14]) via a longitudinal midline aperture in the abdominal wall. This aperture closes over to form the umbilicus, which, in most extant reptiles (including birds), persists for only a few days to weeks and is therefore a common indicator of an individual’s relative maturity (e.g., [15–17]). In late-stage embryonic birds, the umbilicus forms a circular aperture in the abdominal wall that is reduced to a small circular scab that typically drops off within a few days of hatching (Fig. 2E; Additional file 1: Table S1; see also Fig. 5 in Kenny and Cambre [18]). Although scarring does not normally occur, it may persist in some adult birds (e.g., rock pigeon, *Columba livia* [19]) as a linear scar on the lower abdominal wall [19]. Scarring in lepidosaurs (snakes and lizards) is not obvious because of the presence of scales; however, the position of the umbilicus in the adults of some species of snake is retained as a faint line or crease in the ventral scales [15] (Fig. 2B; Additional file 1: Table S2). In lizards, scarring is typically absent but the umbilicus may be demarcated and clearly recognized by a change in abdominal scale morphology. This is typically reflected as a longitudinal row of paired scales that are generally larger and of a different shape to the surrounding abdominal scales and which remains visible throughout life, very similar to the condition in *Psittacosaurus* (Fig. 2; Additional file 1: Table S2). Between the paired scales, a clear line of interstitial tissue delimits the midline and the former umbilical aperture (Fig. 2A), which is observable also in *Psittacosaurus* even where the paired scales more closely match the size and shape of the surrounding abdominal scales (Fig. 1B, C). Such a distinct line of interstitial tissue is not visible anywhere else on the abdomen of SMF R 4970. Where they do occur in modern lizards, paired scale rows are often moderately long and linear features (approx. 7% of SNV; Additional file 1: Table S2), although we have also observed some as long as 11% of the total snout-vent length (SVL) (e.g., spiny-tailed lizard, *Uromastix* sp.; Fig. 2A; Additional file 1: Table S2)—which is similar in size to that of SMF R 4970 (14% of SNV) and to late-stage embryonic birds (approx. 13% of SNV; Additional file 1: Table S2). These values contrast with the relatively shorter umbilici in snakes (approx. 2% of SNV; Additional file 1: Table S2), which is influenced by the elongation of the trunk region. Among crocodylians, the umbilicus is generally absent in adults but remains distinct in *Alligator mississippiensis*, forming an elongate, spiderweb-like arrangement of small scales between the transverse bands of larger quadrangular scales [20]. This arrangement is unique to *Alligator mississippiensis* [20] although it is variably present in other crocodylians as a more subdued line in some individuals with or without marginal paired scales (Fig. 2D). In both crocodylians and lepidosaurs that lose their umbilicus, the pattern of abdominal scales remains undisturbed along the midline (Figs. 2F and 3). Based on the close structural and topographic similarity, particularly to some crocodylians and squamates, we interpret the ventral midline structure in *Psittacosaurus* SMF R 4970 as delimiting the umbilicus, which remained visible for many years, at least into sexual maturity (6+ years).

Where present, the persistence of umbilical scarring into adulthood among reptiles and birds may be species-specific and its position even sexually dimorphic in some cases [15]. Scarring may also be linked to yolk sac infections (omphalitis) in farmed birds and crocodylians, which is connected to improper conditions pre- or post-hatching (e.g., hygiene, diet, temperature/humidity imbalance), although these adverse conditions are rarely present in the wild [21, 22]. Therefore, the presence of an umbilical scar in *Psittacosaurus* does not imply its presence in mature (or near-mature) individuals of all non-avian dinosaur species, although we cannot reject this possibility at this time.

The regular sizes, smooth margins and disposition of the umbilical scales along the midline of *Psittacosaurus*—which is similar to those in wild-caught reptiles (Additional file 1: Table S2)—as well as the absence of regenerative tissue, all rule out a traumatic or pathological origin [21]. Because of the haphazard way in which they are inflicted, traumatic injuries, in contrast, can truncate/bisect individual scales and heal by forming granulation tissue—a type of smooth, scale-free connective tissue—over the open wound [23–25]. In reptiles, scales may not regrow, leaving a central region of the smooth dermis that may be surrounded by a disrupted region of small, irregular scales that differ from the surrounding ‘normal’ scales [23, 24]. Trauma-induced granulation tissue surrounded by a ring of disrupted scales has been reported in the skin of at least one dinosaur [26], but none of these characteristic features are observable in SMF R 4970.

Remnants of the soft tissues associated with embryogenesis and early post-hatching development (including the umbilicus) are rarely captured in the fossil record. To our knowledge, an umbilical scar has not been previously reported in any fossil amniote [27], although the ‘umbilical cord’ was described in a Devonian-aged viviparous placoderm fish [28]. Embryonic skin has been reported in only two non-avian dinosaurs [29–31], neither of which show indications of an embryonic umbilicus, and few other specimens preserve naked integument in this region of the abdomen that can be compared with SMF R 4970. Two unique specimens that do—the hadrosaurid *Edmontosaurus annectens* ‘mummy’ AMNH FARB 5060 and the juvenile ankylosaurian *Liaoningosaurus paradoxus* IVPP V12560—show no indication of any structure that could be interpreted as an umbilicus, even though the latter was less than a year old at the time of death [32]. The skin of *Liaoningosaurus* [33], however, does not perfectly intersect with the midline of the animal and the umbilicus, if present, may simply have not been preserved. The linear row of paired quadrangular scales in an embryonic titanosaur from the famous Auca Mahuevo locality is from an unknown position on the body that was presumed to be along the dorsal midline [30]. Although a ventral midline position is also tenable, an umbilical origin for this structure in the Auca Mahuevo titanosaurs can be rejected as the animals were still *in ovo*, during which time the abdominal aperture would still have been open.

## Conclusions

The remarkable Senckenberg specimen of *Psittacosaurus* sp. (SMF R 4970) was first reported in 2002 and continues to yield surprising details that have been pivotal for reconstructing dinosaurian soft tissues. We add to these details our identification of the umbilical scar, which is the first reported in non-avian dinosaurs and the oldest record of this structure in any fossil amniote. In *Psittacosaurus,* the umbilical scar is recognised by a distinctive linear arrangement of paired abdominal scales that surrounds the former attachment site of the yolk sac. This configuration is consistent with the umbilicus of some modern reptiles (lizards and crocodylians) but differs morphologically from those of snakes and birds, which either lack paired scales or lack scales altogether. While umbilical scarring is normally lost within days to weeks in extant reptiles and birds, persistent scarring into adulthood can be species-specific or the result of individual variation. This discovery provides direct insight into embryonic physiology and the development of a non-avian dinosaur after its connection to the yolk sac was severed.

## Methods

### Laser-stimulated fluorescence (LSF)

Specimen SMF R 4970 was examined first hand and photographed by M.P. and T.G.K. in 2016 using an updated version of the methodology proposed by Kaye et al. [34] and refined in Wang et al. [35]. A 405-nm violet near-UV laser diode was used to fluoresce the specimen following standard laser safety protocol. Long exposure images were taken with a Nikon D810 DSLR camera fitted with a 425-nm blocking filter and controlled from a laptop using *digiCamControl*. Image post-processing (equalization, saturation and colour balance) was performed uniformly across the entire field of view in Adobe Photoshop CS6. Because soft tissue outlines in SMF R 4970 are best visible using LSF, the observations made using this technique and the resulting digital images formed the basis for the descriptions in this study. All measurements were taken using digital images uploaded and calibrated in ImageJ v1.52q.

### Comparative specimens

For comparison with SMF R 4970, a representative sample of snakes, lizards, birds and crocodylians were observed in the collections of the University of New England’s Natural History Museum (Armidale, Australia) (see Additional file 1: Table S2) and supplemented by the primary literature. Additional avian umbilicus observations were made on privately owned domestic chickens. Specimens were photographed using an Olympus S7X7 stereomicroscope fitted with an Olympus SC50 digital camera. Multifocal image stacks were manually captured using cellSens Standard (www.olympus-lifescience.com) imaging software and stacked in Adobe Photoshop CC 2019.

## Supplementary Information

**Additional file 1: TableS1**. Selected scale measurements taken from the abdomen of *Psittacosaurus* SMF R4970. **TableS2**. Observations and measurements of umbilical scarring in representative extant snakes, lizards, crocodylians, and birds.

## Data Availability

*Psittacosaurus* sp. specimen SMF R 4970 is on public display in the Dinosaurs Unlimited permanent exhibition at the Senckenberg Research Institute and Natural History Museum Frankfurt, Frankfurt, Germany (Forschungsinstitut Senckenberg, Frankfurt am Main, Germany). There is ongoing debate regarding the legal ownership of this specimen and efforts to repatriate it to China have not been successful [36]. Our international team of Australian, Belgian, British, Chinese and American members all hope for and support an amicable solution to this ongoing debate. We think it is important to note that the specimen was acquired by the Senckenberg Museum to prevent its sale into private hands and to ensure its availability for scientific study [6]. The specimen is currently inventoried as SMF R 4970 in the museum’s collection and remains available to qualified researchers for scientific study [6]. The datasets supporting the conclusions of this article are included within the article and Additional file 1.
