# Supplementary material for: Oldest preserved umbilical scar reveals dinosaurs had ‘belly buttons’
Source: BMC Biol. 2022 Jun 7;20:132. doi: 10.1186/s12915-022-01329-9 (PMC9172161; doi:10.1186/s12915-022-01329-9)
Supplement: Supplementary file 1 — Additional file 1: TableS1. Selected scale measurements taken from the abdomen of Psittacosaurus SMF R4970. TableS2. Observations and measurements of umbilical scarring in representative extant snakes, lizards, crocodylians, and birds. [file 12915_2022_1329_MOESM1_ESM.docx]

**Additional File 1.**

Oldest preserved umbilical scar reveals dinosaurs had ‘belly buttons’.

Phil R. Bell, Christophe Hendrickx, Michael Pittman, and Thomas G. Kaye

**Table S1.** Selected scale measurements taken from the abdomen of *Psittacosaurus* SMF R4970. Numbers in bold are averages.

| Abdominal scar | Abdomen (post.) | Abdomen (ant.) |
| --- | --- | --- |
| Length (mm) | Length (mm) | Length (mm) |
| 2.3 | 1.1 | 1.8 |
| 2.2 | 1.2 | 1.8 |
| 2.7 | 1.5 | 1.8 |
| 2.8 | 1.5 | 1.3 |
| 2.4 | 1.8 | 2.2 |
| **2.4** | **1.42** | **1.78** |

**Table S2.** Observations and measurements of umbilical scarring in representative extant snakes, lizards, crocodylians, and birds. SVL, snout-vent length; UL, umbilical length; UNE = Natural History Museum of the University of New England, Armidale, NSW, Australia.

| **UNE Accession No.** | **Taxon** | **Genus/Species** | **Common Name** | **Umbilical form** | **SVL (mm)** | **UL (mm)** | **UL as a % of SVL** | **Preservation** |
| --- | --- | --- | --- | --- | --- | --- | --- | --- |
| UNE NR 1614 | Agamidae | *Amphibolurus barbatus* | Eastern Water Dragon | Faint longitudinal line between scales | 185.8 | 13.3 | 7.2 | Wet specimen |
| UNE NENH RE-00030 |  | *Pogona barbata* | Bearded dragon | Faint longitudinal line between scales | 135.2 | 10.7 | 7.9 | Dried specimen |
| UNE NENH RE-00145 |  | *Moloch horridus* | Thorny Devil | Prominent longitudinal line with paired quadrangular scales around margin | 85.7 | 6.3 | 7.4 | Wet specimen |
| UNE NENH RE-00137 |  | *Ctenophorus nuchalis* | Central Netted Dragon | Faint longitudinal line between scales | 107.1 | 8.1 | 7.6 | Wet specimen |
| UNE NENH RE-00147 |  | *Uromastix sp.* | Spiny-tailed lizard | Prominent longitudinal line with paired quadrangular scales around margin | 164.1 | 18.2 | 11.1 | Wet specimen |
| UNE NENH RE-00206 | Scincidae | *Tiliqua scincoides* | Blue-tongued lizard | Immature individual; No umbilicus; uninterrupted overlapping scales across entire abdomen | 157.2 | - | - | Wet specimen |
| UNE no number |  | *Bellatorias major* | Land mullet | No umbilicus; uninterrupted overlapping scales across entire abdomen | 264.0 | - | - | Wet specimen |
| UNE NENH RE-00021 | Lacertidae | *Lacerta viridis* | European Green Lizard | Enlarged polygonal paired scales along entire ventrum - no clear umbilicus | 99.7 | 38.0 | 38.1 | Dried specimen |
| UNE X CB 1 | Crocodylia | *Crocodylus porosus* | Saltwater Croc | (neonate) longitudinal split between abdominal scales, soft scaleless skin, yolk sac still attached | 112.7 | 31.4 | 31.4 | Wet specimen |
| UNE NENH RE-00004 |  | *Crocodylus johnsoni* | Freshwater croc | (<1 yr old) no evidence of umbilicus | 288 | - | - | Dried specimen |
| UNE XBm26 | Serpentes | *Notechis scutatus* |  | (neonate) Split in ventral scales | 251.2 | 4.2 | 1.7 | Wet specimen |
| UNE XBm42 |  | *Pseudonaja textilis* | Eastern brown snake | (neonate) Split in ventral scales | 241.3 | 3.5 | 1.5 | Wet specimen |
| UNE XB 16 |  | *Lampropelitis getulus* |  | (neonate) Fold in ventral scales | 226.5 | 6.4 | 2.8 | Wet specimen |
| UNE XI Hb5 | Aves | *Gallus gallus domesticus* | Domestic chicken | (18 day old embryo) Yolk sac still attached; circular aperture in abdominal wall | 79.6 | 10.4 | 13.1 | Wet specimen |
| UNE XI Hb6 |  | *Gallus gallus domesticus* | Domestic chicken | (17 day old embryo) Yolk sac still attached; circular aperture in abdominal wall | 76.4 | 9.3 | 12.2 | Wet specimen |
